# Risk factors for stroke recurrence in patients with hemorrhagic stroke

**DOI:** 10.1038/s41598-022-22090-7

**Published:** 2022-10-13

**Authors:** Yi-Sin Wong, Ching-Fang Tsai, Cheung-Ter Ong

**Affiliations:** 1 Department of Family Medicine, Ditmanson Medical Foundation Chiayi Christian Hospital, Chiayi City, Taiwan; 2 Department of Nursing, Min-Hwei Junior College of Health Care Management, Tainan, Taiwan; 3 Department of Medical Research, Ditmanson Medical Foundation Chiayi Christian Hospital, Chiayi City, Taiwan; 4 Department of Neurology, Ditmanson Medical Foundation Chiayi Christian Hospital, 539 Chung-Shao Road, Chiayi City, Taiwan

**Keywords:** Diseases, Medical research, Neurology, Risk factors

## Abstract

The risk factors for recurrence of hemorrhagic or ischemic stroke in patients with intracranial hemorrhage (ICH) are inconclusive. This study was designed to investigate the risk factors for stroke recurrence and the impact of antiplatelet on stroke recurrence in patients with ICH. This population-based case-cohort study analyzed the data obtained from a randomized sample of 2 million subjects in the Taiwan National Health Insurance Research Database. The survival of patients with hemorrhagic stroke from January 1, 2000, to December 31, 2013, was included in the study. During the 5-year follow-up period, the recurrence rate of stroke was 13.1% (7.01% hemorrhagic stroke, and 6.12% ischemic stroke). The recurrence rate of stroke was 13.3% in the without antiplatelet group and 12.6% in the antiplatelet group. The risk factor for hemorrhagic stroke was hypertension (OR 1.87). The risk factors for ischemic stroke were age (OR 2.99), diabetes mellitus (OR 1.28), hypertension (OR 2.68), atrial fibrillation (OR 1.97), cardiovascular disease (OR 1.42), and ischemic stroke history (OR 1.68). Antiplatelet may decrease risk of hemorrhagic stroke (OR 0.53). The risk of stroke recurrence is high in patients with ICH. Hypertension is a risk factor for ischemic and hemorrhagic stroke recurrence. Antiplatelet therapy does not decrease risk of ischemic stroke recurrence but may reduce recurrence of hemorrhagic stroke.

## Introduction

Though the incidence and mortality rates of stroke with intracranial hemorrhage (ICH) declined gradually^1^, hemorrhagic stroke still accounts for 10–20% of patients with stroke^2–4^. The global burden of hemorrhagic stroke is gradually increasing. Risk factors for ICH included hypertension and warfarin use^5–7^. The survival outcomes after hemorrhagic stroke include mortality, functional impairment, quality of life impairment, intracerebral hemorrhage recurrence, ischemic stroke, cognitive impairment, psychiatric diseases, and epilepsy^8^. The annual rate of stroke recurrence after ICH was approximately 5–10%^9,10^. However, the report on the type of recurrent stroke after the patients with ICH is inconclusive. Studies have reported that patients with ICH have a higher risk of hemorrhage recurrence than those with ischemic stroke^11,12^. Some studies have reported that patients with deep ICH have a higher risk of ischemic stroke recurrence than those with hemorrhagic stroke and those with lobar hemorrhage have an increased risk of hemorrhagic stroke recurrence^10^. However, some studies have found that the type of recurrent stroke was not significantly different between patients with lobar hemorrhage and those with deep hemorrhage stroke^9,13^. Murthy et al. have found that compared with individuals without ICH, patients with ICH have an increased risk of ischemic stroke with a hazard ratio (HR) of 3.1^14^.

A study has shown that inadequate blood pressure control increases the probability of ICH recurrence^15^. In patients with ICH who received antiplatelet drugs, the rate of ICH recurrence and hemorrhage volume did not increase^16,17^. In contrast, Biffi et al. have found that the use of antiplatelet drugs may increase the risk of ICH recurrence in patients with lobar hemorrhage^15^.

Stroke recurrence may increase mortality and impair daily activity and function. Presently, the risk factors for ischemic and hemorrhagic stroke recurrence remain inconclusive. Therefore, this study was designed to determine and investigate the risk factors for ischemic or hemorrhagic stroke recurrence and the impact of antiplatelet on stroke recurrence in patients with ICH in Taiwan.

## Methods

### Data source and ethics approval

This population-based case-cohort study used data from the Taiwan National Health Insurance Research Database (NHIRD) that comprises data obtained from 2 million individuals. The National Health Insurance (NHI) program in Taiwan has been operating since 1995. The NHIRD is a research database developed by the NHI Research Institute and contains patient healthcare data from hospitals, outpatient clinics, and community pharmacies. NHI encompasses more than 99% of 23 million individuals and 95% of the hospitals in Taiwan. The NHI Research Institute provides the database to researchers after anonymizing all personal information. This study included data retrieved from the “Longitudinal Health Insurance Database” (LHID 2005) from a random sample of 2 million individuals within the NHIRD, with linked longitudinal data available from 2000 to 2013. The randomized data (LHID 2005) represent all beneficiaries as no significant differences in sex, age, and premium rate are found between individuals in the LHID 2005 and the original NHIRD datasets. The codes of the International Classification of Diseases, Ninth Revision (ICD-9) were used to define diseases. This study was approved by the Institutional Review Board of Ditmanson Medical Foundation Chiayi Christian Hospital, Taiwan (CYCH-IRB: 2020131). Due to the anonymity of the NHIRD, the requirement for informed consent from each patients was waived by the IRB of Ditmanson Medical Foundation Chiayi Christian Hospital.

### Study subjects and definitions

Hemorrhagic stroke was defined as an episode of rapidly developing neurological deficit attributed to the focal collection of blood within the brain parenchyma or ventricular system (ICH) or due to subarachnoid bleeding (subarachnoid hemorrhage) not caused by trauma^18^. All hospitalized patients with the diagnosis of hemorrhagic stroke (ICD-9 codes: 430 and 431) between January 1, 2000 and December 31, 2013 were included in the study. Recurrent hemorrhagic stroke was defined as patient re-hospitalization with a discharge diagnosis of hemorrhagic stroke (ICD-9 codes: 430 and 431). Recurrent ischemic stroke was defined as patient re-hospitalization with a discharge diagnosis of ischemic stroke (ICD-9 codes, 433, 434, and 436). Stroke risk factors were defined as the following disease diagnosis during discharge in patients with hemorrhagic stroke: hypertension (ICD-9 code, 401, 405), diabetes mellitus (ICD-9 code, 250, 648), atrial fibrillation (ICD-9 code, 427), cardiovascular disease (ICD-9 code, 492, 648), congestive heart failure (ICD-9 code, 402, 404, 428), and dyslipidemia (ICD-9 code, 272). All patients underwent computed tomography or magnetic resonance imaging to confirm the occurrence of stroke. The exclusion criteria were as follows: (1) patients younger than 20 years; (2) those who had a history of hemorrhagic stroke; (3) those who continue to use antiplatelet drugs for more than 7 days 1 month before the onset of hemorrhagic stroke; (4) those with stroke recurrence within 1 month before hemorrhagic stroke onset; and (5) those who died within 1 month after stroke onset.

**Figure 1:**
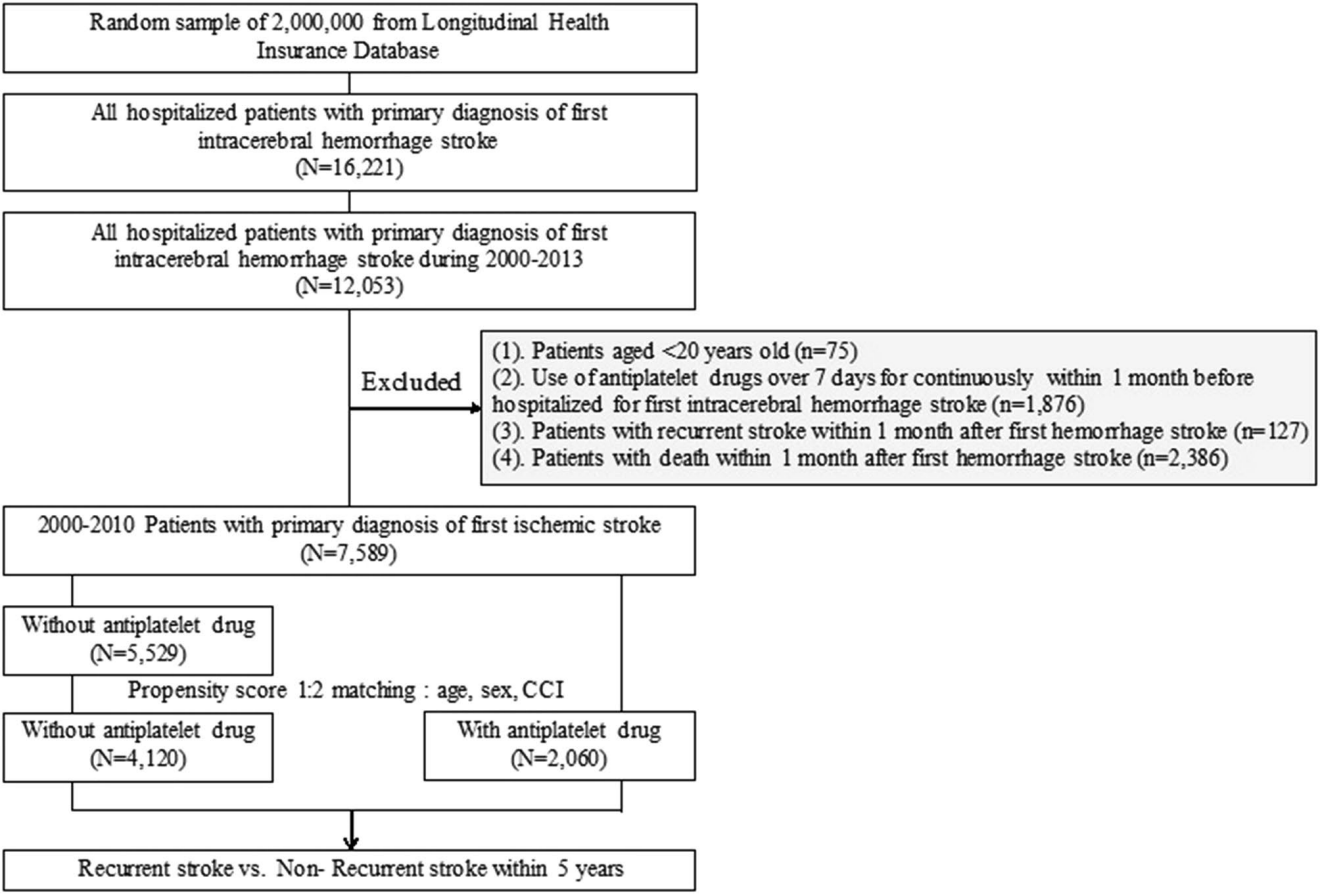
Flow chart of patient enrollment. CCI: clinical comorbidity index.

In total, 12,053 patients were hospitalized under the discharge diagnosis of hemorrhagic stroke. Among them, 75 patients were younger than 20 years, 1876 patients used antiplatelet drugs for more than 7 days within 1 month before stroke onset, 127 patients had stroke recurrence within 1 month after stroke onset, and 2386 patients died within 1 month after stroke onset, all of whom were excluded from this study. Finally, 7589 patients were included in the study (Fig. 1). The patients who continued to use one or two of the drugs—aspirin, ticlopidine, and clopidogrel—were classified in the use antiplatelet group.

Separate propensity score matches were performed using a ratio of 1:2 to compare the risk of stroke recurrence between the groups with and without the use of antiplatelet drugs. All methods were performed in accordance with the relevant guidelines and regulations.

### Outcome measure

The primary endpoint of this study was new-onset hemorrhagic or ischemic stroke.

### Statistical analysis

In this study, we compared the rate of stroke recurrence between patients who used antiplatelet drugs and those who did not receive antiplatelet drugs using propensity score matching with a ratio of 1:2 to match the study patients based on age, sex, and clinical comorbidity index (CCI). The baseline characteristics of the patients as categorical variables were compared using the chi-square test. Continuous variables were compared using the t-test. Cox proportional hazards regression models were used to evaluate the HRs and 95% confidence intervals (CIs) for the recurrence of ischemic and hemorrhagic stroke in each risk factor. All statistical analyses were performed using SAS statistical software (version 9.4; SAS Institute, Inc, Cary, NC). *P* < 0.05 was considered statistically significant.

## Results

From 2000 to 2013, 7589 patients fulfilled the inclusion criteria for this study. The characteristics of the 7589 patients are shown in Table 1. After 1:2 propensity score matching for sex, age, and CCI, 6180 patients were included in the analysis, including 4120 patients in the without antiplatelet group and 2060 patients in the antiplatelet group. The area under the curve of the propensity score was 0.544. After propensity score matching, age, sex, stroke severity, and ischemic stroke history were not significantly different between the without antiplatelet and antiplatelet groups. The probability of admission to the intensive care unit and hospitalization day longer than 9 days was higher in the without antiplatelet group. The rates of diabetes mellitus, hypertension, hyperlipidemia, atrial fibrillation, cardiovascular disease, and congestive heart failure were higher in the antiplatelet group (Table S1).

**Table 1:** Characteristics of the patients with hemorrhagic stroke (n = 7589).

Variables	Without antiplatelet (n = 5529)	Antiplatelet (n = 2060)	*P*
**Age**			
20–44	832 (15.05)	138 (6.70)	< 0.0001
45–59	1960 (35.45)	744 (36.12)	
≥ 60	2737 (49.50)	1178 (57.18)	
Mean ± SD	60.09 ± 15.16	62.27 ± 12.31	
**Sex**			
Female	2176 (39.36)	802 (38.93)	0.7365
Male	3353 (60.64)	1258 (61.07)	
**Length of stay, days**			
< 9	1236 (22.35)	595 (28.88)	< 0.0001
≥ 9	4293 (77.65)	1465 (71.12)	
**ICU**			
No	4294 (77.66)	1742 (84.56)	< 0.0001
Yes	1235 (22.34)	318 (15.44)	
**CCI, mean ± SD**			
< 5	3711 (67.12)	1318 (63.98)	0.0101
≥ 5	1818 (32.88)	742 (36.02)	
Mean ± SD	3.96 ± 2.76	4.16 ± 2.72	0.0040
**Diabetes mellitus**			
No	3917 (70.84)	1334 (64.76)	< 0.0001
Yes	1612 (29.16)	726 (35.24)	
**Hypertension**			
No	751 (13.58)	91 (4.42)	< 0.0001
Yes	4778 (86.42)	1969 (95.58)	
**Hyperlipidemia**			
No	3955 (71.53)	1295 (62.86)	< 0.0001
Yes	1574 (28.47)	765 (37.14)	
**AF**			
No	5332 (96.44)	1937 (94.03)	< 0.0001
Yes	197 (3.56)	123 (5.97)	
**CVD**			
No	4289 (77.57)	1298 (63.01)	< 0.0001
Yes	1240 (22.43)	762 (36.99)	
**CHF**			
No	3250 (58.78)	914 (44.37)	< 0.0001
Yes	2279 (41.22)	1146 (55.63)	
**The history of IS**			
No	5314 (96.11)	1958 (95.05)	0.0396
Yes	215 (3.89)	102 (4.95)	
**Recurrent stroke**			
No	4792 (86.67)	1800 (87.38)	< 0.0001
Yes	737 (13.33)	260 (12.62)	
IS	306 (5.53)	159 (7.72)	0.0004
Hemorrhagic stroke	431 (7.79)	101 (4.90)	< 0.0001

ICU, intensive care unit; CCI, clinical comorbidity index; AF, atrial fibrillation; CHF, congestive heart failure; IS, ischemic stroke; SD, standard deviation; CVD, cardiovascular disease.

During the 5-year follow-up period, stroke in 997 patients recurred; the recurrence rate of stroke was 13.1% (997/7589), of whom 7.01% (532/7589) had hemorrhagic stroke and 6.12% (465/7589) had ischemic stroke. The rate of stroke recurrence was 13.3% (737/5529) in the without antiplatelet group and 12.6% (260/2060) in the antiplatelet group. Moreover, the rate of hemorrhagic stroke recurrence was lower in the antiplatelet group and the rate of ischemic stroke recurrence was higher in the antiplatelet group. The recurrence rates of hemorrhagic stroke were 7.79% (431/5529) and 4.9% (101/2060) in the without antiplatelet and antiplatelet groups (*P* < 0.01), while the recurrence rates of ischemic stroke were5.53% (306/5529) and 7.72% (159/2060) (*P* < 0.01) respectively.

The risk factors for ischemic stroke recurrence were age, sex, diabetes mellitus, hypertension, atrial fibrillation, cardiovascular disease, and ischemic stroke history. The HR of ischemic stroke was 2.17 (95% CI 1.38–3.41) in patients aged between 45 and 59 years and 2.99 (95% CI 1.91–4.68) in patients aged ≥ 60 years. The HR of ischemic stroke was 1.28 (95% CI 1.04–1.57) in patients with diabetes mellitus, 2.68 (95% CI 1.55–4.63) in patients with hypertension, 1.97 (95% CI 1.40–2.76) in patients with atrial fibrillation, 1.42 (95% CI 1.15–1.74) in patients with cardiovascular disease, and 1.69 (95% CI 1.18–2.40) in patients with a history of ischemic stroke. The antiplatelet group did not reduce risk of ischemic stroke with an HR of 0.90 (95% CI 0.74–1.09) (Table 2).

**Table 2:** The relationship between antiplatelet agent use and 5-year rate of ischemic stroke recurrence among patients with first-time hemorrhage stroke (n = 7589).

Variables	Number	Number of IS event	Person-years	Incidence rate per 100 person-years	Crude HR (95% CI)	Adjusted HR (95% CI)
**Age**						
20–44	970	22	4259.6	0.52	Ref	Ref
45–59	2704	157	11,294.0	1.39	2.68 (1.71–4.18)	2.17 (1.38–3.41)
≥ 60	3915	286	13,318.7	2.15	4.05 (2.62–6.24)	2.99 (1.91–4.68)
**Sex**						
Female	2978	171	11,381.1	1.50	Ref	Ref
Male	4611	294	17,491.3	1.68	1.12 (0.93–1.35)	1.23 (1.02–1.49)
**Length of stay, days**						
< 9	1831	140	7485.4	1.87	Ref	Ref
≥ 9	5758	325	21,386.9	1.52	0.81 (0.66–0.99)	0.90 (0.73–1.11)
**ICU**						
No	6036	399	23,614.7	1.69	Ref	Ref
Yes	1553	66	5257.6	1.26	0.74 (0.57–0.96)	0.86 (0.76–0.97)
**CCI**						
< 5	5029	295	20,121.6	1.47	Ref	Ref
≥ 5	2560	170	8750.7	1.94	1.29 (1.07–1.56)	0.87 (0.70–1.08)
**Antiplatelet agents**						
No	5529	306	19,990.5	1.53	Ref	Ref
Yes	2060	159	8881.8	1.79	1.18 (0.97–1.43)	0.90 (0.74–1.09)
**Diabetes mellitus**						
No	5251	286	20,459.9	1.40	Ref	Ref
Yes	2338	179	8412.4	2.13	1.50 (1.25–1.81)	1.28 (1.04–1.57)
**Hypertension**						
No	842	14	3237.6	0.43	Ref	Ref
Yes	6747	451	25,634.7	1.76	4.02 (2.36–6.84)	2.68 (1.55–4.63)
**Hyperlipidemia**						
No	5250	287	19,453.8	1.48	Ref	Ref
Yes	2339	178	9418.5	1.89	1.29 (1.07–1.55)	1.11 (0.92–1.35)
**AF**						
No	7269	425	27,910.2	1.52	Ref	Ref
Yes	320	40	962.1	4.16	2.64 (1.91–3.65)	1.97 (1.40–2.76)
**CVD**						
No	5587	291	21,742.8	1.34	Ref	Ref
Yes	2002	174	7129.5	2.44	1.80 (1.49–2.17)	1.42 (1.15–1.74)
**CHF**						
No	4164	220	16,216.6	1.36	Ref	Ref
Yes	3425	245	12,655.8	1.94	1.41 (1.18–1.69)	0.98 (0.81–1.20)
**The history of IS**						
No	7272	429	27,966.0	1.53	Ref	Ref
Yes	317	36	906.3	3.97	2.51 (1.79–3.53)	1.69 (1.18–2.40)

ICU, intensive care unit; CI, confidence interval; AF, atrial fibrillation; CHF, congestive heart failure; IS, ischemic stroke; HR, hazard ratio; CVD, cardiovascular disease.

The factors for hemorrhagic stroke recurrence included hypertension. The HR of hemorrhagic stroke was 1.87 (95% CI 1.32–2.64) in patients with hypertension. After adjusting for possible confounding factors (age, sex, stroke severity, diabetes mellitus, hypertension, hyperlipidemia, atrial fibrillation, cerebrovascular disease, congestive heart failure, and ischemic stroke history), the use of antiplatelet drugs was found to decrease the risk of hemorrhagic stroke with an HR of 0.53 (95% CI 0.42–0.66) (Table 3).

**Table 3:** The relationships between antiplatelet agent use and 5-year rate of hemorrhagic stroke recurrence among patients with first-time hemorrhagic stroke (n = 7589).

Variables	Number	Number of HS event	Person-years	Incidence rate per 100 person-years	Crude HR (95% CI)	Adjusted HR (95% CI)
**Age**						
20–44	970	70	4259.6	1.64	Ref	Ref
45–59	2704	202	11,294.0	1.79	1.07 (0.82–1.41)	0.93 (0.70–1.23)
≥ 60	3915	260	13,318.7	1.95	1.11 (0.86–1.45)	1.09 (0.90–1.32)
**Sex**						
Female	2978	208	11,381.1	1.83	Ref	Ref
Male	4611	324	17,491.3	1.85	1.01 (0.85–1.20)	1.00 (0.84–1.19)
**Length of stay, days**						
< 9	1831	134	7485.4	1.79	Ref	Ref
≥ 9	5758	398	21,386.9	1.86	1.03 (0.84–1.25)	1.00 (0.82–1.23)
**ICU**						
No	6036	427	23,614.7	1.81	Ref	Ref
Yes	1553	105	5257.6	2.00	1.08 (0.87–1.34)	0.99 (0.93–1.06)
**CCI**						
< 5	5029	392	20,121.6	1.95	Ref	Ref
≥ 5	2560	140	8750.7	1.60	0.78 (0.65–0.95)	0.79 (0.64–0.98)
**Antiplatelet agents**						
No	5529	431	19,990.5	2.16	Ref	Ref
Yes	2060	101	8881.8	1.14	0.54 (0.44–0.67)	0.53 (0.42–0.66)
**Diabetes mellitus**						
No	5251	390	20,459.9	1.91	Ref	Ref
Yes	2338	142	8412.4	1.69	0.86 (0.71–1.05)	0.97 (0.79–1.20)
**Hypertension**						
No	842	39	3237.6	1.20	Ref	Ref
Yes	6747	493	25,634.7	1.92	1.57 (1.13–2.17)	1.87 (1.32–2.64)
**Hyperlipidemia**						
No	5250	392	19,453.8	2.02	Ref	Ref
Yes	2339	140	9418.5	1.49	0.75 (0.62–0.91)	0.78 (0.64–0.96)
**AF**						
No	7269	510	27,910.2	1.83	Ref	Ref
Yes	320	22	962.1	2.29	1.16 (0.76–1.78)	1.32 (0.85–2.04)
**CVD**						
No	5587	419	21,742.8	1.93	Ref	Ref
Yes	2002	113	7129.5	1.58	0.80 (0.65–0.99)	0.89 (0.71–1.11)
**CHF**						
No	4164	307	16,216.6	1.89	Ref	Ref
Yes	3425	225	12,655.8	1.78	0.92 (0.78–1.09)	0.96 (0.80–1.16)
**The history of IS**						
No	7272	513	27,966.0	1.83	Ref	Ref
Yes	317	19	906.3	2.10	1.06 (0.67–1.67)	1.14 (0.72–1.82)

ICU, intensive care unit; CI, confidence interval; AF, atrial fibrillation; CHF, congestive heart failure; IS, ischemic stroke; HS, hemorrhagic stroke; CVD, cardiovascular disease.

When considering factors of sex, age, and stroke severity, we analyzed the risk factors of stroke recurrence in patients post propensity score matching (n = 6180).

The risk factors for ischemic stroke recurrence were age, sex, diabetes mellitus, hypertension, atrial fibrillation, cardiovascular disease, and ischemic stroke history. The HR of ischemic stroke was 1.98 (95% CI 1.10–3.57) in patients aged between 45 and 59 years and 2.49 (95% CI 1.38–4.47) in patients aged ≥ 60 years. The HR of ischemic stroke was 2.24 (95% CI 1.27–3.93) in patients with hypertension, 1.87 (95% CI 1.30–2.71) in patients with atrial fibrillation, 1.42 (95% CI 1.15–1.77) in patients with cardiovascular disease, and 1.56 (95% CI 1.06–2.30) in those with a history of ischemic stroke. The antiplatelet group did not reduce the risk of ischemic stroke with an HR of 0.80 (95% CI 0.70–1.05) (Table 4).

**Table 4:** The relationship between antiplatelet agent use and 5-year rate of ischemic stroke recurrence among patients with first-time hemorrhagic stroke (n = 6180).

Variables	Number	Number of IS event	Person-years	Incidence rate per 100 person-years	Crude HR (95% CI)	Adjusted HR (95% CI)
**Age**						
20–44	405	12	1782.4	0.67	Ref	Ref
45–59	2319	149	9696.5	1.54	2.27 (1.26–4.09)	1.98 (1.10–3.57)
≥ 60	3456	260	12,180.0	2.13	3.10 (1.75–5.53)	2.49 (1.38–4.47)
**Sex**						
Female	2415	156	9258.4	1.68	Ref	Ref
Male	3765	265	14,400.5	1.84	1.09 (0.90–1.33)	1.18 (0.96–1.45)
**Length of stay, days**						
< 9	1502	129	6133.4	2.10	Ref	Ref
≥ 9	4678	292	17,525.5	1.67	0.79 (0.64–0.97)	0.87 (0.7–1.09)
ICU						
No	4945	364	19,444.9	1.87	Ref	Ref
Yes	1235	57	4214.0	1.35	0.72 (0.54–0.95)	0.86 (0.76–0.97)
**CCI**						
< 5	4002	265	16,027.4	1.65	Ref	Ref
≥ 5	2178	156	7631.4	2.04	1.21 (0.99–1.48)	0.93 (0.74–1.17)
**Antiplatelet agents**						
No	4120	262	14,777.1	1.77	Ref	Ref
Yes	2060	159	8881.8	1.79	1.02 (0.84–1.24)	0.8 (0.7–1.05)
**Diabetes mellitus**						
No	4161	261	16,263.2	1.60	Ref	Ref
Yes	2019	160	7395.7	2.16	1.34 (1.10–1.63)	1.21 (0.97–1.50)
**Hypertension**						
No	523	13	1944.2	0.67	Ref	Ref
Yes	5657	408	21,714.7	1.88	2.79 (1.60–4.84)	2.24 (1.27–3.93)
**Hyperlipidemia**						
No	4185	264	15,572.2	1.70	Ref	Ref
Yes	1995	157	8086.7	1.94	1.15 (0.95–1.40)	1.03 (0.84–1.27)
**AF**						
No	5919	388	22,843.5	1.70	Ref	Ref
Yes	261	33	815.3	4.05	2.32 (1.63–3.31)	1.87 (1.30–2.71)
**CVD**						
No	4455	263	17,368.4	1.51	Ref	Ref
Yes	1725	158	6290.5	2.51	1.64 (1.35–2.00)	1.42 (1.15–1.77)
**CHF**						
No	3267	197	12,734.5	1.55	Ref	Ref
Yes	2913	224	10,924.4	2.05	1.32 (1.09–1.59)	1.01 (0.82–1.24)
**The history of IS**						
No	5905	391	22,855.6	1.71	Ref	Ref
Yes	275	30	803.3	3.73	2.13 (1.47–3.09)	1.56 (1.06–2.30)

ICU, intensive care unit; CI, confidence interval; AF, atrial fibrillation; CHF, congestive heart failure; IS, ischemic stroke; HR, hazard ratio; CVD, cardiovascular disease.

The factors for hemorrhagic stroke recurrence included hypertension. The HR of hemorrhagic stroke was 1.87 (95% CI 1.32–2.64) in patients with hypertension. The use of antiplatelet drugs was found to decrease the risk of hemorrhagic stroke with an HR of 0.52 (95% CI 0.42–0.66) (Table 5).

**Table 5:** The relationships between antiplatelet agent use and 5-year rate of hemorrhagic stroke recurrence among patients with first-time hemorrhagic stroke (n = 6180).

Variables	Number	Number of HS event	Person-years	Incidence rate per 100 person-years	Crude HR (95% CI)	Adjusted HR (95% CI)
**Age**						
20–44	405	30	1782.4	1.68	Ref	Ref
45–59	2319	165	9696.5	1.70	1.00 (0.68–1.48)	1.10 (0.74–1.63)
≥ 60	3456	260	12,180.0	1.90	1.07 (0.73–1.57)	1.10 (0.89–1.35)
**Sex**						
Female	2415	156	9258.4	1.84	Ref	Ref
Male	3765	265	14,400.5	1.78	0.97 (0.80–1.18)	0.95 (0.78–1.15)
**Length of stay, days**						
< 9	1502	129	6133.4	1.87	Ref	Ref
≥ 9	4678	292	17,525.5	1.77	0.94 (0.76–1.16)	0.90 (0.72–1.12)
**ICU**						
No	4945	364	19,444.9	1.78	Ref	Ref
Yes	1235	57	4214.0	1.90	1.05 (0.82–1.34)	1.01 (0.95–1.07)
**CCI**						
< 5	4002	265	16,027.4	1.90	Ref	Ref
≥ 5	2178	156	7631.4	1.59	0.80 (0.65–0.99)	0.82 (0.65–1.04)
**Antiplatelet agents**						
No	4120	262	14,777.1	2.20	Ref	Ref
Yes	2060	159	8881.8	1.14	0.53 (0.42–0.66)	0.52 (0.42–0.66)
**Diabetes mellitus**						
No	4161	261	16,263.2	1.86	Ref	Ref
Yes	2019	160	7395.7	1.68	0.89 (0.72–1.09)	0.98 (0.78–1.23)
**Hypertension**						
No	523	13	1944.2	1.08	Ref	Ref
Yes	5657	408	21,714.7	1.87	1.71 (1.10–2.65)	2.05 (1.31–3.22)
**Hyperlipidemia**						
No	4185	264	15,572.2	1.96	Ref	Ref
Yes	1995	157	8086.7	1.50	0.78 (0.63–0.96)	0.81 (0.65–1.01)
**AF**						
No	5919	388	22,843.5	1.78	Ref	Ref
Yes	261	33	815.3	2.33	1.24 (0.78–1.96)	1.40 (0.87–2.24)
**CVD**						
No	4455	263	17,368.4	1.91	Ref	Ref
Yes	1725	158	6290.5	1.51	0.78 (0.62–0.98)	0.86 (0.67–1.10)
**CHF**						
No	3267	197	12,734.5	1.85	Ref	Ref
Yes	2913	224	10,924.4	1.75	0.94 (0.77–1.13)	0.98 (0.80–1.20)
**The history of IS**						
No	5905	391	22,855.6	1.78	Ref	Ref
Yes	275	30	803.3	2.37	1.24 (0.79–1.97)	1.35 (0.84–2.17)

ICU, intensive care unit; CI, confidence interval; AF, atrial fibrillation; CHF, congestive heart failure; IS, ischemic stroke; HS, hemorrhagic stroke; CVD, cardiovascular disease.

## Discussion

The main findings of this study include the following: (1) in patients with ICH stroke, the 5-year rate of stroke recurrence was 13.1%; (2) stroke recurrence was more frequent in patients with hemorrhagic stroke than that in patients with ischemic stroke with a ratio of 1.13; (3) antiplatelet therapy cannot decrease the risk of ischemic stroke but may decrease the risk of hemorrhagic stroke recurrence; (4) hypertension may increase the risk of hemorrhagic and ischemic stroke recurrence.

Studies have shown that in patients with ICH stroke who were followed for less than 3–5 years, the rate of hemorrhagic stroke recurrence was between 7 and 12%. In a single-center study, with a mean follow-up period of less than 3.6 years, Hill et al. have found that the rate of hemorrhagic stroke recurrence was 8.72% (15/172)^10^. Bailey et al. reviewed data with a mean follow-up of 3.4 person-year and found that the rate of hemorrhagic stroke recurrence was 8.35% (157/1880) and 2.4% per patient-year^11^. In a study with a follow-up duration of less than 5 years, Vermeer has reported that the rate of hemorrhagic stroke recurrence was 12% (30/243) and the annual rate of ICH recurrence was 2.1%^19^. In this study, with a follow-up duration of less than 5 years, the rate of hemorrhagic stroke recurrence was 7.01%, with incidence 1.64–1.95 patient-years in each age group, which is mildly lower than that reported in a previous study. The difference may be due to the stroke type. Studies have shown that patients with lobar hemorrhage have a higher risk of hemorrhage recurrence than those with deep hemorrhage^9,11,12^. Vermeer has reported a higher rate of hemorrhagic stroke recurrence than this study; the difference may be related to the higher proportion (55%) of patients with lobar hemorrhage in Vermeer’s study^19^.

The rate of ischemic stroke recurrence was 6.12% in patients who did not use antiplatelet drugs. The rate of ischemic stroke recurrence was close to that reported by Vermeer et al.^19^ but lower than that found in another study^13^. In a study with a 5-year follow-up period, Casolla et al. have found that the cumulative rate of ischemic stroke recurrence was 9.8%. The difference could be because the patients in this study was younger than those in Casolla et al.’s study (mean age, 62.2 years vs. 70 years, respectively), and this study included patients with subarachnoid hemorrhage. Moreover, patients with hemorrhagic stroke have a higher risk of ischemic stroke recurrence than those without stroke history. Murthy et al. have found that patients with a history of hemorrhagic stroke had a higher risk of ischemic stroke recurrence than those without hemorrhagic stroke history, with an HR of 3.1^14^.

Some studies have found that patients with hemorrhagic stroke have a higher risk of brain hemorrhage than those with ischemic stroke^11^. However, some studies have found that ischemic stroke is more frequent than hemorrhagic stroke^9,10,13^. Our study found that hemorrhagic stroke was more frequent than ischemic stroke. The difference may be related to patient characteristics and location of ICH. Studies have found that patients with lobar hemorrhage have a higher risk of ICH recurrence, and deep hemorrhage increases the risk of ischemic stroke recurrence^10,20^. ICH is also a risk factor for ischemic stroke recurrence.

It is reasonable to consider that antiplatelet therapy may increase the risk of hemorrhage and decrease the risk of ischemic stroke. However, this study found that antiplatelet therapy did not decrease the risk of ischemic stroke but may decrease the risk of hemorrhagic stroke. The result agrees with those reported in a previous study. Murthy et al. have found that antiplatelet therapy did not affect mortality and clinical outcomes in patients with ICH^21^. The condition may be because most patients with hemorrhagic stroke have many stroke risk factors, which may affect the recurrence of ischemic and hemorrhagic stroke (Table 1).

The study found that hypertension may increase the risk of hemorrhagic and ischemic stroke recurrence. Age, atrial fibrillation, cardiovascular disease, and history of ischemic stroke are also risk factors of ischemic stroke recurrence. Intensive control blood pressure may decrease the risk of ischemic and hemorrhagic stroke recurrence.

This study has several limitations. First, this was a retrospective study; we did not have data regarding deep hemorrhage or lobar hemorrhage. Second, stroke recurrence was based on the diagnosis of ischemic or hemorrhagic stroke among hospitalized patients, which may have underestimated the rate of stroke recurrence. Third, information on stroke risk control, such as blood pressure, blood sugar, and blood lipid level, was unavailable, which may have affected the rate of stroke recurrence and stroke type. Fourth, this is a retrospective observational study, we cannot rule out the possibility that antiplatelet agent were prescribed to the patient whom less likely to have recurrence of hemorrhagic stroke.

In conclusion, the risk of stroke recurrence is high in patients with ICH. Antiplatelet therapy does not decrease risk of ischemic stroke recurrence but may decrease risk of hemorrhagic stroke recurrence. Hypertension is a risk factor of ischemic and hemorrhagic stroke recurrence.

## Supplementary Information

Supplementary Information.

## Data Availability

All data generated or analysed during this study are included in this published article and its supplementary information files.
