# Supplementary material for: Risk factors for stroke recurrence in patients with hemorrhagic stroke
Source: Sci Rep. 2022 Oct 13;12:17151. doi: 10.1038/s41598-022-22090-7 (PMC9562220; doi:10.1038/s41598-022-22090-7)
Supplement: Supplementary file 1 — Supplementary Information. [file 41598_2022_22090_MOESM1_ESM.docx]

Table S1. Characteristics of the patients with hemorrhagic stroke (n=6180)

| Variables | Without antiplatelet | Antiplatelet | P |
| --- | --- | --- | --- |
| Age |  |  |  |
| 20–44 | 267 (6.48) | 138 (6.70) | 0.27 |
| 45–59 | 1 575 (38.23) | 744 (36.12) |  |
| ≥60 | 2278 (55.29) | 1178 (57.18) |  |
| Mean ± SD | 62.21 ± 12.59 | 62.27 ± 12.31 |  |
| Sex |  |  |  |
| Female | 1613 (39.15) | 802 (38.93) | 0.86 |
| Male | 2507 (60.85) | 1258 (61.07) |  |
| Length of stay, days |  |  |  |
| <9 | 907 (22.01) | 595 (28.88) | <0.01 |
| ≥9 | 3213 (77.99) | 1465 (71.12) |  |
| ICU |  |  |  |
| No | 3203 (77.74) | 1742 (84.56) | <0.01 |
| Yes | 917 (22.26) | 318 (15.44) |  |
| CCI, mean ± SD | 4.08 ± 2.70 | 4.16 ± 2.72 | 0.27 |
| Diabetes mellitus |  |  |  |
| No | 2827 (68.62) | 1334 (64.76) | <0.01 |
| Yes | 1293 (31.38) | 726 (35.24) |  |
| Hypertension |  |  |  |
| No | 432 (10.49) | 91 (4.42) | <0.01 |
| Yes | 3688 (89.51) | 1969 (95.58) |  |
| Hyperlipidemia |  |  |  |
| No | 2890 (70.15) | 1295 (62.86) | <0.01 |
| Yes | 1230 (29.85) | 765 (37.14) |  |
| AF |  |  |  |
| No | 3982 (96.65) | 1937 (94.03) | <0.01 |
| Yes | 138 (3.35) | 123 (5.97) |  |
| CVD |  |  |  |
| No | 3157 (76.63) | 1298 (63.01) | <0.01 |
| Yes | 963 (23.37) | 762 (36.99) |  |
| CHF |  |  |  |
| No | 2353 (57.11) | 914 (44.37) | <0.01 |
| Yes | 1767 (42.89) | 1146 (55.63) |  |
| The history of IS |  |  |  |
| No | 3947 (95.80) | 1958 (95.05) | 0.17 |
| Yes | 173 (4.20) | 102 (4.95) |  |
| Recurrent stroke |  |  |  |
| No | 3533 (85.75) | 1800 (87.38) | <0.01 |
| Yes | 587 (14.25) | 260 (12.62) |  |
| IS | 262 (6.36) | 159 (7.72) | 0.04 |
| Hemorrhagic stroke | 325 (7.89) | 101 (4.90) | <0.01 |

ICU, intensive care unit; CCI, clinical comorbidity index; AF, atrial fibrillation;

CHF, congestive heart failure; IS, ischemic stroke; SD, standard deviation;

CVD, cardiovascular disease.
